# Analysis of ultra-deep targeted sequencing reveals mutation burden is associated with gender and clinical outcome in lung adenocarcinoma

**DOI:** 10.18632/oncotarget.8213

**Published:** 2016-03-19

**Authors:** Dakai Xiao, Hui Pan, Fuqiang Li, Kui Wu, Xin Zhang, Jianxing He

**Affiliations:** ^1^ Department of Thoracic Surgery, The First Affiliated Hospital of Guangzhou Medical University, Guangzhou, China; ^2^ Research Center for Translational Medicine, The First Affiliated Hospital of Guangzhou Medical University, Guangzhou, China; ^3^ Guangzhou Institute of Respiratory Disease and State Key Laboratory of Respiratory Disease, Guangzhou, China; ^4^ BGI-Shenzhen, Shenzhen, China

**Keywords:** lung adenocarcinoma, targeted NGS sequencing, mutation burden

## Abstract

Gender-associated difference in incidence and clinical outcomes of lung cancer have been established, but the biological mechanisms underlying these gender-associated differences are less studied. Recently we have characterized the genomic landscape of lung adenocarcinoma derived from Chinese population (Reference [1]). In this study we evaluated the clinical significance of mutation burden in lung adenocarcinoma and found that the male tumors harbored statistically greater burden of genetic alterations than female counterparts (Male median 3 (range 0–34) vs female median = 2 (0–24), male to female ratio = 1.636, 95% CI = 1.343–1.992) after adjustment of age at surgery, stage, smoking status. Kaplan-Meier survival analysis revealed that greater burden of genetic alterations was associated with worse overall survival. Moreover, multivariable analysis demonstrated mutation burden was an independent prognostic factor for the patients. Taken together, our analysis demonstrated gender disparity of mutation burden and their prognostic value in lung adenocarcinoma. This gender difference in mutation burden might provide an explanation for the distinct difference in the clinical outcomes between sexes in lung adenocarcinoma.

## INTRODUCTION

Lung cancer is the most frequently diagnosed cancer and leading cause of cancer-related death among men. In contrast, among women lung cancer is the third most frequently diagnosed cancer and second leading cause of cancer–related death worldwide [2]. There were estimated 1,241,600 newly diagnosed cases of lung cancer among men and 583,100 newly diagnosed cases among women, and moreover men accounted for 70% of lung cancer-related deaths [2]. The gender-associated difference in clinical outcome of lung cancer has also been well confirmed in several population-based studies [3–6]. Although the gender difference in lung cancer is diminishing in North American and some European countries, this difference remains in other areas [7]. Such gender disparities in incidence and clinical outcomes of lung cancer have been attributed to the differences in smoking habit, environmental exposure, genetic variants, lifestyle and sex hormones activity between sexes [8, 9]. However, the biological mechanisms underlying the gender-associated differences in incidence and clinical outcomes are still less explored.

With the advances in cancer genomic sequencing, cancer is considered as a genomic disease resulting from progressive accumulation of genetic aberrations in the process of initiation and dissemination of tumor, and a small proportion of mutations have been identified as potential driver mutations, which are likely associated with initiation and progression of cancer [10–12]. Recently we have characterized the genetic landscape of lung adenocarcinoma in Chinese population and identified the significantly altered genes in this lethal disease using whole genome or whole exome sequencing [1]. Based-on the results from genomic sequencing, we thus developed a customized cancer panel targeting 51 genes which are closely associated with lung cancer. Although aberrations in well-known driver genes have been linked to clinical outcomes of patients, the prognostic value of molecular genotyping is limited by the low prevalence of some driver mutations [13]. Since the progression of cancer is accompanied by the accumulation of distinct genetic alterations, we test if the mutation burden in the primary tumors could predict the prognosis of the patients with lung adenocarcinoma. In this study, we found that male tumors harbored higher burden of genetic alterations than female counterparts, and greater burden of genetic alterations was also associated with worse clinical outcomes. The gender-associated difference in mutation burden will help gain insight into the biological mechanism of gender disparity in clinical outcome of lung adenocarcinoma.

With the increasing number of patients whose tumors are sequenced with targeted sequencing technology, this finding will provide the prognostic value of sequencing data other than choosing suitable targeted therapy for the patients with lung adenocarcinoma.

## RESULTS

### Mutational signature in lung adenocarcinoma patients

Recently we have characterized the genetic landscape of lung adenocarcinoma using whole-genome or whole exome sequencing. In the discovery set, we identified a set of genes that are recurrently mutated in Chinese patients with lung adenocarcinoma [1]. To further confirm the mutated genes in lung adenocarcinoma patients, we performed a hybrid-recapture and ultra-deep DNA sequencing on a set of 51 selected genes that were closely associated with the progression of lung cancer (Gene list was available in Supplementary Table S1).

**Figure 1:**
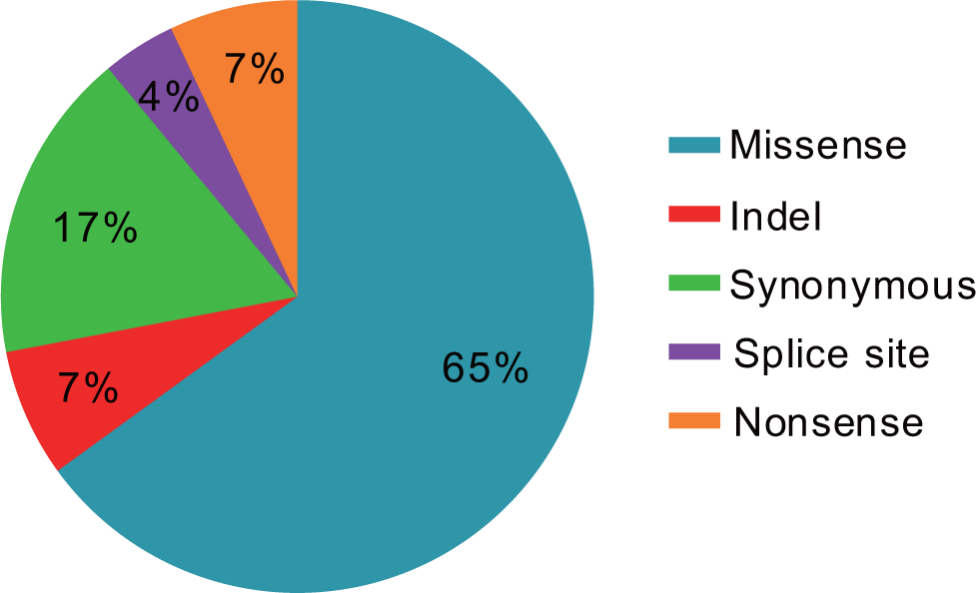
Pie chart showing the different types of genetic alterations found in 335 patients with lung adenocarcinoma

Through the ultra-deep targeted sequencing, we identified total 962 alterations from 335 cases including missense, frameshift and synonymous mutations (Figure 1), among which, missense mutation (632/962, 65%) was the major type of genetic alterations. The median number of alterations per patient was 2 (range, 0–34). Most of patients (293/335,87.5%) had at least one alteration. Forty-two patients (12.5%) had no alteration, 79 patients (23.6%) had one alteration, 74 patients (22.1%) had 2 alterations, 66 patients (19.7%) had 3 alterations, and 74 patients (22.1%) had 4 or more alterations (Table 1).

**Table 1:** The frequency of genetic alterations (total mutation and missense mutations) identified in 335 patients with lung adenocarcinoma

Number of Total mutations	Number of patients (%)	Number of missense mutations	Number of patients (%)
0	42 (12.54)	0	72 (21.49)
1	79 (23.58)	1	98 (29.25)
2	74 (22.09)	2	86 (25.67)
3	66 (19.70)	3	40 (11.94)
4	27 (8.06)	> 3	39 (11.64)
> 4	47 (14.03)		

### The association of mutation burden with clinical features in lung adenocarcinoma patients

Targeted sequencing for a set of cancer genes is a strategy to screen actionable targets for the individual patients. In our cohort, clinical significance of individual genetic alteration was evaluated, only variants in TP53, STK11, BRAF, MET, LRP1B and MRC2 were found to be associated with worse overall survival [1]. However, the predictive value of these genes is limited by the low frequency of these genetic alterations.

Besides identifying actionable targets, we also tried to determine the clinical significance of mutation burden in lung adenocarcinoma with analysis of targeted sequencing data. To determine the association of mutation burden with clinical feature of lung adenocarcinoma, we firstly analyzed the number of genetic alterations in different groups stratified according to clinical features such as age at surgery, gender, TNM stage and smoking status. Univariate analysis with negative binomial regression model showed that number of genetic alterations was statistically significantly associated with gender and smoking status (Table 2). Male tumors harbored more total alterations than female tumors (Table 2, male median 3 (range 0–34) vs female median 2 (range 0–24), Male to female ratio = 1.636, 95% CI = 1.343–1.992). And tumors from smokers also had more total genetic alterations than those from non-smokers (Table 2, Smokers median 3 (range 0–34) vs non-smokers median 2 (range 0–32), *p* = 0.00139). Further multivariate analysis revealed that male tumors harbored statistically significantly higher burden of genetic alterations than female counterparts (Table 2, Multivariable male to female ratio = 1.524, 95% CI = 1.189–1.956) even after adjustment of age at surgery, stage, smoking status while there was no significant difference in mutation burden between smokers and non-smokers. Interestingly, as missense mutation was the major type of genetic alteration in our cohort, we also found that the male tumors harbored greater burden of missense mutation than female tumors after adjusting for other clinical features(Supplementary Table S2).

**Table 2:** Univariate and multivariate analysis with negative binomial regression comparing the mutation counts by variables in patients with lung adenocarcinoma (*n* = 335)

	No. of patients	No. of total mutations median (range)	Univariate analysis		Multivariate analysis	
			***p*** value	Male-to-Female Ratio	***p*** value	Male-to-Female Ratio
Gender				(95%CI Ratio)		(95%CI Ratio)
Female	152	2 (0–24)	(reference)	1.636 (1.343–1.992)	(reference)	1.524 (1.189~1.956)
Male	183	3 (0–34)	**9.64E–07**		**0.000819**	
Age						
< 65	228	2 (0–34)	(reference)		(reference)	
≥ 65	107	2 (0–32)	0.616		0.381	
Smoking						
No	199	2 (0–32)	(reference)		(reference)	
Yes	105	3 (0–34)	**0.00139**		0.361	
NA	31					
Stage						
I	82	2 (0–32)	(reference)		(reference)	
II	69	2 (0–22)	0.378		0.691	
III	154	2 (0–24)	0.893		0.532	
IV	29	2(0–34)	0.541		0.317	
NA	1					

NA, not applicable.

Tobacco smoking is a mutagen for lung cancer, and most of smokers were male in our cohort. To further test whether the gender-associated difference in mutation burden was independent of smoking status, we also compared the number of genetic alterations between sexes among non-smokers. This subgroup analysis showed that in non-smokers, male tumors also harbored more total alterations (Table 3. male to female ratio = 1.486, 95% CI = 1.124–1.968, *p* = 0.0055)) and missense mutations (Supplementary Table S3. Male-to-female ratio = 1.481, 95% CI = 1.099–1.996, *p* = 0.0099) than female tumors. However, among smokers, there was no statistically difference in mutation burden between sexes probably due to the limited size of female smokers (*n* = 9) (Tables 3 and S3). Taken together, these results indicated that there was a greater burden of genetic alterations among men compared to women, and also suggested tobacco smoking exposure alone could not fully explain the gender–associated difference in mutation burden. Other exogenous or endogenous factors might be involved in the mutational processes.

**Table 3:** Negative binomial regression comparing the counts of total mutations in smokers and non-smokers with lung adenocarcinoma

		No. of patients	No. of total mutations Median (Range)	*p* value	Male to female ratio (95% CI ratio)
Non-smoker	Female	128	2 (0–24)	(reference)	1.486 (1.124~1.968)
	Male	71	3 (0–32)	**5.52E–03**	
Smoker	Female	9	2 (1–5)	(reference)	1.496 (0.824–2.733)
	Male	96	3 (0–34)	0.18562	

### The clinical significance of mutation burden in lung adenocarcinoma

**Figure 2:**
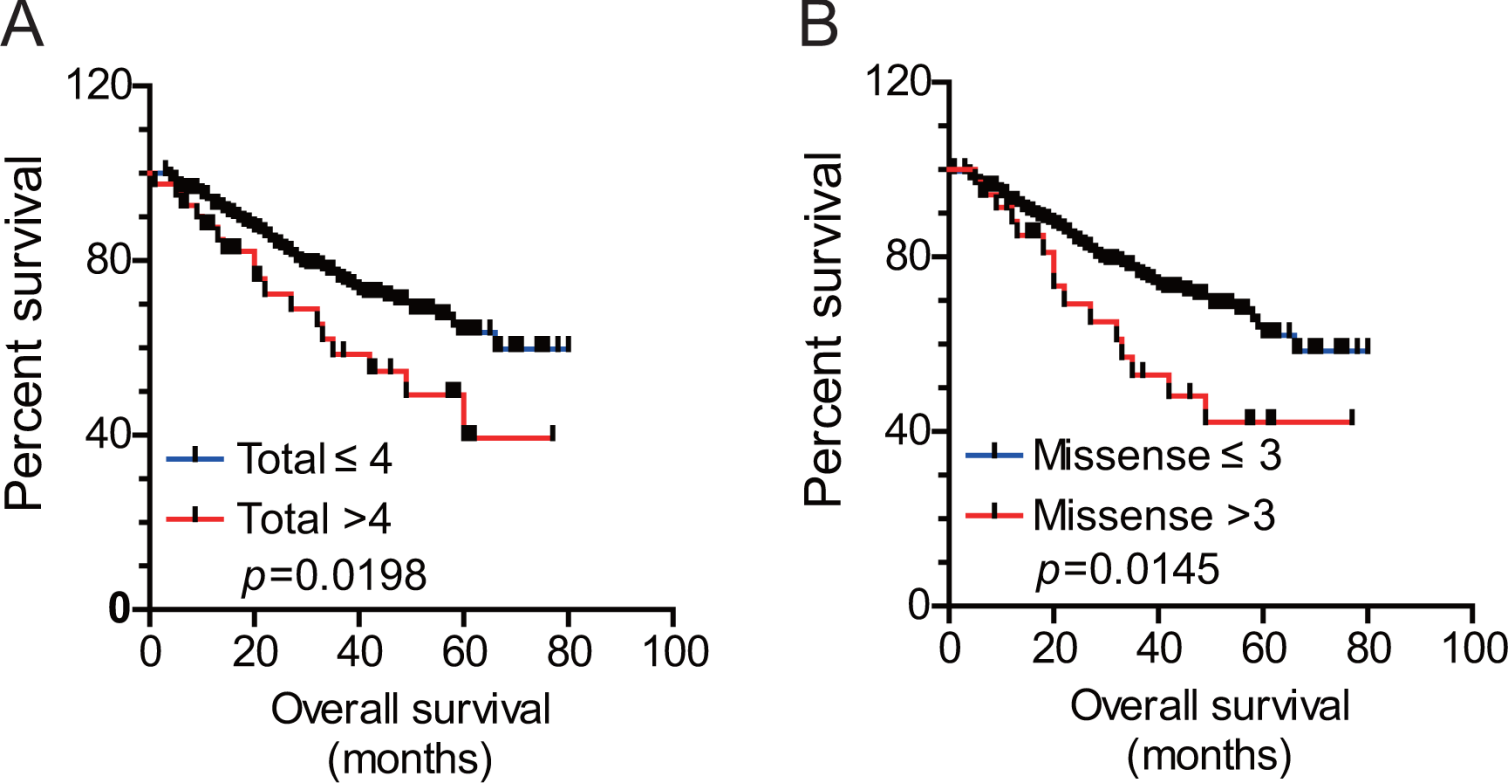
The burden of genetic alterations was associated with overall survival of the lung adenocarcinoma patients Kaplan-Meier overall survival curves for patients with higher or lower burden of total mutations or missense mutation were shown in Figure 2A and 2B, respectively.

To investigate the clinical significance of mutation burden in lung adenocarcinoma patients, we performed Kaplan-Meier survival analysis and revealed that the patients whose tumors harbored greater burden of genetic alterations (> 4 total genetic alterations) was correlated with poorer overall survival (Figure 2A, log rank test, *p* = 0.0198). The mean survival time for the patients whose tumor harbored ≤ 4 and > 4 total alterations was 61 months and 48 months, respectively (Table 4). Of note, we also found the greater burden of missense mutations (> 3 missense mutations), was also associated with worse overall survival (Figure 2B, log rank test, *p* = 0.0145). The mean survival time for the patients whose tumor harbored ≤ 3 and > 3 missense mutations was 61 months and 47 months, respectively. These data suggested that mutation burden was associated with overall survival of the lung adenocarcinoma patients.

**Table 4:** Univariate and multivariate survival analysis of the patients with lung adenocarcinoma (*n* = 335)

	Mean Survival (months)	Univariate analysis			Multivariate Analysis		
		HR	95% CI	*p* value	HR	95% CI	*p* value
Age < 65 *vs* ≥ 65	59.0 *vs* 58.0	0.87	0.55~1.36	0.538	1.20	0.76~1.88	0.429
Gender female *vs* male	63.0 *vs* 55.0	1.71	1.12~2.61	0.013	2.04	1.24~3.35	0.005
Smoking No *vs* Yes	59.5 *vs* 56.2	0.98	0.62~1.57	0.947	0.69	0.40~1.17	0.384
Stage				< 0.0001			< 0.0001
I	69.1				(Reference)		
II	54.8				3.03	1.41~6.51	
III	56.5				3.95	1.96~7.98	
IV	31.4				8.76	3.86~19.9	
Total mutation ≤ 4 *vs* > 4	61.0 *vs* 48.4	2.19	1.13~4.24	0.020	1.81	1.05~3.13	0.034

In addition, univariate survival analysis with log rank test also showed that TNM stage and gender were correlated with overall survival (Table 4, *p* < 0.001 and *p* = 0.013, respectively), while smoking status was not associated with survival of the lung adenocarcinoma patients (Table 4). To examine the impact of the mutation burden on the survival of smokers and non-smokers, we also performed Kaplan-Meier analysis of mutation burden in smokers and non-smokers, respectively. The subanalyses revealed that higher burden of genetic alterations were tended to be correlated with worse overall survival at the margin of statistical significance among non-smokers and smokers (Figure S1A–S1D).

To further determine the prognostic value of mutation burden, we thus performed a multivariable analysis with Cox proportional hazard regression model. The results showed total genetic alterations > 4 (Table 4, HR = 1.81, 95% CI = 1.05–3.13, *p* = 0.034) was an independent risk factor for the clinical outcome of the patients with lung adenocarcinoma, and similarly, greater burden of missense mutation was also an independent risk factor for the survival (Supplementary Table S4, HR = 1.99, 95% CI = 1.12–3.52, *p* = 0.019). These results suggested that higher burden of genetic alterations represented an independent risk factor for the lung adenocarcinoma patients. Furthermore, consistent with previous reports [3–6, 14], female patients with lung adenocarcinoma had a better overall survival than male patients (Table 4, mean survival 63.0 vs 55.0 months for women and men, respectively, *p* = 0.013), and gender was an independent prognostic factor for lung adenocarcinoma patients (Table 4, HR = 2.04, 95% CI = 1.24~3.35, *p* = 0.005).

## DISCUSSION

In present study we characterized the burden of genetic alterations and assessed their prognostic value in patients with lung adenocarcinoma and found that there was different mutation burden between male and female tumors, and higher burden of genetic alterations was associated with worse clinical outcome. We also showed the evidence to support that mutation burden is an independent prognostic factor for lung adenocarcinoma patients. These analyses demonstrated for the first time a gender difference in burden of recurrent mutation in lung adenocarcinoma. This finding also provided a biological explanation for gender difference in clinical outcome of lung adenocarcinoma in our cohort.

In this study, the mutation burden was obtained through ultra-deep targeted sequencing in a set of selected genes rather than missense mutation obtained through whole genomic sequencing. There are several reasons we focused on the burden of these selected genes: first, 1) although thousands of mutations have been identified in cancer genome sequencing, most of these mutations are ‘passengers’ [15], only a handful was recognized as driver mutations which are believed to be associated with cancer progression. 2) mutation recurrence has proven to be a powerful tool for the identification of cancer gene [16]. Most of genes in this panel have been identified to be recurrently mutated in Chinese population, suggesting that these mutations are highly potential drivers, not passenger mutations in cancer progression. 3) Targeted sequencing of a panel of cancer genes can be rapidly performed in several sequencing platform and has been applied to estimate the mutational burden [17].

It is interesting to note that Gupta et al. [18] also found the gender-associated difference in mutation burden in cutaneous melanoma through the analysis of exomic sequences. However, they found that greater burden of missense mutation was associated with improved survival although men harbored greater burden of mutation and exhibited higher incidence and poorer outcome than women [18]. They provided an ‘immune fitness’ hypothesis to interpret the inconsistency between mutation burden and clinical outcomes. Similar to melanoma, lung adenocarcinoma was also one type of mutagen-induced tumors and harbored highest number of mutations [19, 20], and displayed gender-associated differences in incidence and clinical outcome between sexes. However, Gupta et al. found no statistically significant difference in mutation burden between sexes in lung adenocarcinoma. In contrast, we found a statistically significantly higher burden of mutations among men, and higher mutation burden was correlated with worse overall survival of lung adenocarcinoma, which was also consistent with several previous studies that men have worse clinical outcomes than women [3–6, 14]. We reasoned that the difference between our study and Gupta et al. mainly relied on the type of sequencing data we analyzed. Gupta et al. [18] performed the analysis based on the exome sequencing data, most of missense mutation was considered as passenger mutation, while our analysis was performed mainly based on the genes which were recurrently mutated in lung adenocarcinoma. These genetic alterations were most likely associated with cancer progression while the vast majority of mutations generated from exomic sequences were passenger mutations, which will definitely underestimate the predominant role of driver mutations in cancer progression. These results suggested that we should be cautious to interpret the data considering the different cancer types and cancer panel detected.

It was noteworthy that although we and other group [11] already showed that smokers had more mutations, smoking status was not correlated with the overall survival in our cohort. In fact, inconsistent results about the relationship between clinical outcome and smoking status have been reported [13, 21]. As different mutational processes cause different mutation signatures [16, 19, 22], smokers and non-smoker displayed distinct mutational patterns, we reasoned that in lung adenocarcinoma, somatic mutations could be generated by several mutational processes other than tobacco smoking exposure, such as environmental or occupational exposure, CpG deamination or off-target modification of DNA by APOBEC family which generated distinct mutation signature [16, 23]. Indeed, in our previous study, four highly confident mutational signatures have been extracted from Chinese patients with lung adenocarcinoma. However, which signature contributes to cancer progression and is associated with gender and clinical outcome remains to be investigated in large samples.

Consistent with previous study in Asia [24], we also demonstrated that female patients showed survival advantage over male, and gender was identified as a prognostic factor for survival. Woolston et al. reported lung adenocarcinoma possessed ethnic and gender specific differences in genetic pattern [25]. The female patients smoked much less than male [26], and showed distinct molecular characteristics such as, higher frequency of EGFR alterations, lower frequency of KRAS mutation [27–29] and distinct genetic variants in lung cancer susceptibility loci [30]. Other than these differences, we also showed that female patients in Asia harbored higher burden of mutation than male counterparts. As we also showed that lower burden of recurrent mutation was correlated with improved clinical outcomes, this finding raise the possibility that lower burden of recurrent mutation among women, at least partially, explain the survival advantage in women. However, whether this finding also applied to other ethnic populations is still unknown. Notably, we also found there was no statistically association between mutation burden and stage, which suggested that the detailed mechanisms of mutational process in cancer progression remained to be defined.

This study has similar limitation as described previously [18] such as the targeted sequencing delineated coding sequencing of a set of genes, other genes and non-coding regions were not included. Moreover, the limited sample size of smokers (*n* = 9) among women could decrease the statistical power and diminish the importance of smoking status in survival analysis.

All together, this study demonstrated the gender difference in burden of recurrent mutations which serve as an independent prognostic factor in Chinese patients with lung adenocarcinoma. However, further studies with large sample sizes are needed to clarify whether this finding is also applied to other ethnic populations.

## MATERIALS AND METHODS

### Patient information and specimen collection

This study was approved by the Institutional Review Board of The First Affiliated Hospital of Guangzhou Medical University, and three hundred and thirty-five lung adenocarcinoma patients with informed consent were enrolled in this study. The samples that satisfied the following conditions were included in this study and were subjected to being sequenced as described previously [1]. Firstly, The primary tumors and adjacent normal tissues were surgically resected from lung adenocarcinoma patients and the tumor specimens were then reviewed by independent pathologists to determine the histological subtype, TNM stage and tumor cellularity. Tumors that were not confirmed as adenocarcinoma, or patients with unavailable clinical data, or neoplasms with low tumor cell content (< 50% for primary tumors) were excluded. Secondly, DNA and RNA were extracted from tissues passed clinical evaluation, and quality-control estimation was performed to remove samples of low quality. The demographic information such as gender, age at diagnosis, smoking status and other clinical information were retrieved from the electronic medical record system in our hospital, and the survival status of the patients was followed up over the phone every three months. The patient characteristics were summarized in our previous study [1].

### Data analysis

The mutational profiling for the patients was performed using ultra-deep targeted sequencing and the alterations in the 51 genes for each patient were extracted from our previous study [1]. Negative binomial regression was performed to predict the mutation counts in univariate and multivariable analysis with R package. Pearson's Chi-square was used to compare the association between the mutation burden and gender, age at diagnosis, smoking status. Kaplan-Meier curve was used to estimate the overall survival with log-rank test. Multivariable analysis with Cox proportional hazard regression model was carried out to determine the prognostic significance of mutation burden. The *p* value < 0.05 is considered as statistically significant, and all the statistical tests were two-sided. The analyses were performed using SPSS16.0, R package and GraphPad Prism 6.0.

## SUPPLEMENTARY MATERIALS FIGURE AND TABLES


