# Supplementary material for: Analysis of ultra-deep targeted sequencing reveals mutation burden is associated with gender and clinical outcome in lung adenocarcinoma
Source: Oncotarget. 2016 Mar 19;7(16):22857–64. doi: 10.18632/oncotarget.8213 (PMC5008406; doi:10.18632/oncotarget.8213)
Supplement: Supplementary file 1 [file oncotarget-07-22857-s001.pdf]

Analysis of ultra-deep targeted sequencing reveals mutation burden is associated with gender and clinical outcome in lung adenocarcinoma

Supplementary Materials

Supplementary Table S1: The gene list and number of samples with genetic alterations

Gene Name	No.of samples with genetic alterations	Percentage (%)
TP53	148	44.2
EGFR	134	40.0
LRP1B	62	18.5
KRAS	36	10.7
CDC27	35	10.4
AGGF1	30	9.0
PTPRD	24	7.2
APC	18	5.4
RHPN2	16	4.8
PIK3CA	16	4.8
PARP4	15	4.5
STK11	15	4.5
GLI3	15	4.5
KEAP1	14	4.2
BRAF	13	3.9
ITIH5	12	3.6
ATF7IP	12	3.6
FLT1	11	3.3
IQGAP3	11	3.3
MET	10	3.0
NASP	8	2.4
MRC2	8	2.4
ERBB2	8	2.4
CFTR	7	2.1
SMAD2	6	1.8
CRIPAK	6	1.8
TERT	6	1.8
CDKN2A	6	1.8
CHEK2	6	1.8
SUSD2	6	1.8
RIN3	5	1.5
YES1	4	1.2
TMC6	4	1.2
MECP2	3	0.9

EBF3	3	0.9
FSCN1	3	0.9
PARP1	3	0.9
EML4	3	0.9
MAFB	2	0.6
APOL1	2	0.6
TBP	1	0.3
VEGFA	1	0.3
MAP2K1	1	0.3
NRAS	1	0.3
FADD	0	0.0
NKX2-1	0	0.0
TLX3	0	0.0
CCNE2	0	0.0
AKT1	0	0.0
RANBP10	0	0.0
KDM5B	0	0.0

Supplementary Table S2: Univariate and multivariate analysis with negative binomial regression comparing the counts of missense mutation by variables in patients with lung adenocarcinoma (*n* = 335)

	No. of patients	No. of missense Median (Range)	Univariate analysis		Multivariate analysis	
Gender			*p* value	Male-to-Female Ratio (95% CI Ratio)	*p* value	Male-to-Female Ratio (95% CI Ratio)
Female	152	1 (0–11)	(reference)		(reference)	
Male	183	2 (0–28)	3.49E-05	1.565 (1.267–1.936)	**0.000896**	1.422(1.093~1.852)
Age						
< 65	228	1 (0–15)	(reference)		(reference)	
≥ 65	107	2 (0–28)	0.701		0.997	
Smoking						
No	199	1 (0–28)	(reference)		(reference)	
Yes	105	2 (0–15)	0.0044		0.303	
NA	31					
Stage						
I	82	2 (0–28)	(reference)		(reference)	
II	69	1 (0–11)	0.205		0.587	
III	154	2 (0–7)	0.183		0.652	
IV	29	1 (0–15)	0.947		0.639	
NA	1					

NA, not applicable.

Supplementary Table S3: Negative binomial regression comparing the counts of missense mutations in smokers and non-smokers with lung adenocarcinoma

		No. of patients	No. of missense mutations Median (range)	*p* value	Male to female ratio (95% CI ratio)
Non-smoker	Female	128	1 (0–11)	(reference)	1.481(1.099~1.996)
	Male	71	2 (0–28)	**9.90E-03**	
Smoker	Female	9	2 (1–4)	(reference)	1.151(0.643~2.127)
	Male	96	2 (0–15)	0.6429	

Supplementary Table S4: Univariate and multivariate survival analysis of the patients with lung adenocarcinoma (n = 335)

	Univariate analysis			Multivariate analysis		
	HR	95% CI	*p* value	HR	95% CI	*p* value
Age<br>< 65 vs ≥ 65	0.87	0.55~1.36	0.538	1.19	0.76~1.86	0.452
Gender<br>Female vs Male	1.71	1.12~2.61	0.013	1.97	1.19~3.26	0.008
Smoking<br>No vs Yes	0.98	0.62~1.57	0.947	0.682	0.40~1.17	0.374
Stage		–	< 0.0001			< 0.0001
I				(Reference)		
II				2.93	1.36~6.27	
III				3.74	1.86~7.53	
IV				9.15	4.02~20.8	
Missense mutation<br>≤ 3 vs > 3	2.45	1.19~5.02	0.015	1.99	1.12~3.52	0.019

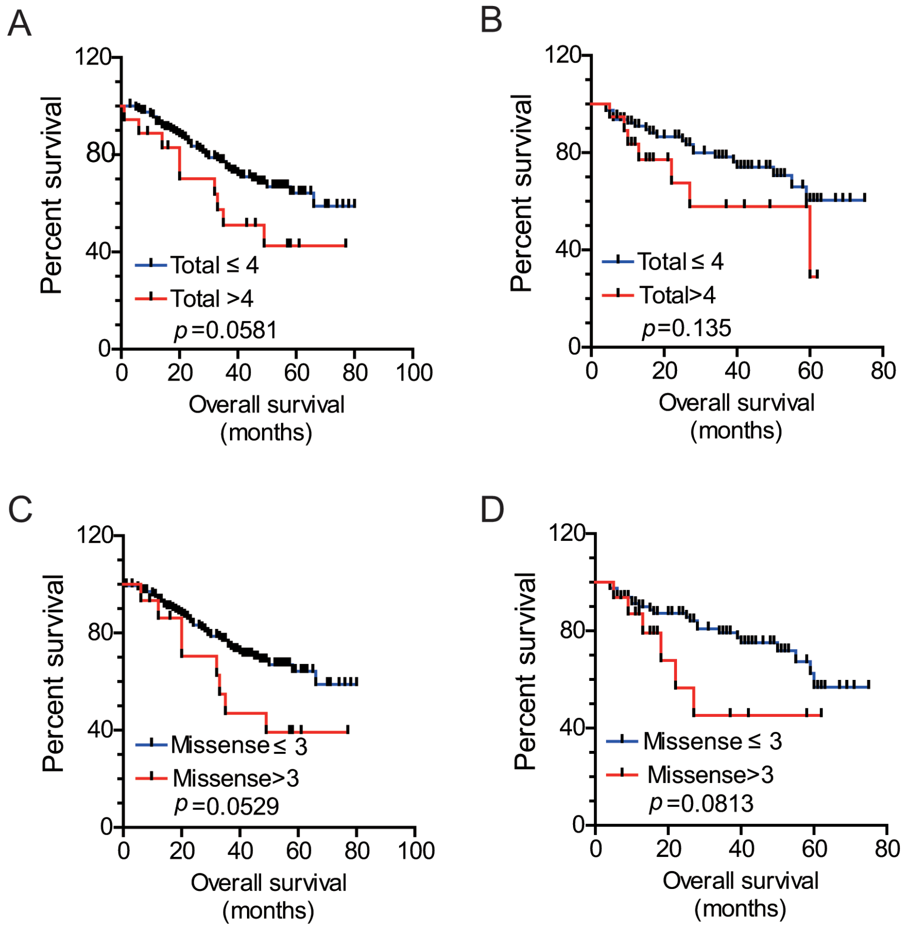

Supplementary Figure S1: Kaplan-Meier overall survival curves for patients with higher or lower burden of total mutations (A and B) or missense mutations (C and D) among non-smokers (A and C) and smokers (B and D) were shown.
